# Annotations

**Published:** 1898-06-04

**Authors:** 


### Annotations.

### English and German Midwifery Teaching.

While English medical students have to wander about the slums of London attending cases of normal labour and hoping that perchance the fates will provide them with something worth seeing during their term of office, often to be disappointed, the Germans arrange matters in a better fashion, at any rate from the educational point of view. In England, if a good case does occur, and if the assistance of the obstetric physician has to be called in the chances are that any operative interference which he undertakes is witnessed only by his assistant and the particular student who happens to be attending, and even then is witnessed under the most unfavourable circumstances, the operation being done, perhaps, in a little room, on a low bed, and in a miserable light. In Buda-Pest, however, the professor of obstetrics has a hospital with 70 obstetric and 40 gynæcological beds, and an amphitheatre for 150 students before whom difficult and instrumental deliveries and obstetric operations are performed. Thus, while on the one hand the operations are done under proper conditions instead of amid the wretched surroundings of English district midwifery, the students all have a clear view of the operation, and can see all that is going on. Again, in Vienna, at the Frauenklinik of the Allgemeine Krankenhaus, an enormous number of confinements take place, so that it is possible to see a very large selection of cases in a short time, and what is much more important, the difficult cases and the operations can be utilised for the teaching of a considerable number of students. It is not in cases, nor in skill, nor in teaching power that London is behind, but in the absolute lack of organisation for the utilisation of its material. While the English student rushes about in the slums and spends his time pacifying the meddlesome women by whom he is surrounded, and furtively impaling the bugs that crawl upon his clothes, the German student sits at ease in a well-lighted theatre and takes notes. No doubt we are backward in many things, but there is nothing in which we are so behind as in the arrangements for the teaching of practical midwifery in London. The London student learns to use his wits, he learns self-reliance and *savoir faire*—but that is not midwifery.

### The Traffic in Dead Meat.

It may, no doubt, be said with a certain degree of truth, that for those who elect to live in crowds, and enjoy the pleasures of great cities, it does not do to be over particular about the sights with which they are surrounded. Nevertheless, we think that any self-respecting community ought to put some limit on what is allowed to be done in the public streets, which, after all, are public property, and ought to exercise some control over the manner of doing things, the doing of which is admittedly a necessity. Now, doubtless, it is necessary to convey meat from one part of the town to another, and, in view of the fact that meat is a perishable article, it probably would be unreasonable to suggest that the transit of it through the streets should be limited to certain hours, as is done in regard to the carriage of certain offensive materials; but surely it is not too much to ask that those engaged in the meat trade should conduct their work in a reasonably decent manner, and in such a way as not to offend the eyes of every passer-by. That meat should be carried through the streets in open carts; that great piles of flesh and fat with bones protruding should be tumbled into common vans and jolted along the stream of ordinary traffic, and that great hulking meat porters should be seen lounging on the top of these piles of meat, smoking and spitting as they go, is an atrocious offence to all delicacy which ought not to be tolerated for a moment by any civilised community. To save the nose we willingly put up with regulations which, by limiting the hours during which manure can be removed, complicate in no inconsiderable degree the sanitary administration of the metropolis. But of the eye we take no note, and we allow these wretched meat porters, with their filthy boots and dirty clothes, not only to injure the meat as an article of diet, but to parade through the streets a nauseating and disgusting exhibition, the toleration of which for a single day reflects nothing but discredit upon our civic rulers. Nor is this all. So utterly unregulated does this trade appear to be that, as we are informed, even cabs are used for the express distribution of meat from the central markets. The cabs which in the early night take our sisters in all their finery to the theatres, are used in the early morning to carry great quarters of greasy meat. In London anything with wheels may be pressed into the service of the meat trade. Needless to say, there are parts of the world in which special vans are employed for the purpose.

### Advertisements and Ethics.

It was rather neat that just before the meeting of the American Medical Editors' Association a facetious advertiser should throw temptation in the way of the medical press of America, and should lead the managers of many journals to stray from the paths of virtue. It happened in this way. The proprietor of a much-advertised patent medicine, who, as is the way with his kind, knew somewhat of human nature, drew up an advertisement which was certainly somewhat of a shocker, and one to try the nerves of any who stickle for propriety in medical journalism. This bait he scattered broadcast before the managers of 119 medical journals in the United States and Canada, and, having had his little joke, he amiably published to the world the names of the journals which swallowed the bait. More than half of the journals took no notice whatever of his communication; a good many declined the advertisement—some with scorn, some with regret; but thirty of them yielded to the tempter, greatly to the amusement of the virtuous remainder. One of the latter says, "It is humiliating to note the avidity with which the bait was gulped down—bob, hook, and sinker—by some editors who boast of the huge circulation and lofty moral tone of their journals." Easy as it is to laugh, however, it is not always so easy to avoid being taken in. Nor is it always easy to fix a standard. We remember more than once—not exactly "when we were boys," but still some time ago—protesting against advertisements which shocked our sense of propriety, only to be faced the following week with copies of other medical journals in which the said advertisements had appeared in full, naked and not at all ashamed. We do what we can, and believe our contemporaries do the same, but to err is human, and everyone must nod sometimes.

